# How to Respond to the US Food and Drug Administration's Report on Squamous Cell Carcinoma in Breast Implant Capsules

**DOI:** 10.1093/asj/sjad088

**Published:** 2023-04-05

**Authors:** Steven Teitelbaum

On September 8, 2022, the US FDA released a safety notification about cases of squamous cell carcinoma (SCC) found in breast implant capsules.^1^ This initially caused great alarm, but subsequent analysis has mitigated panic among most patients and surgeons. There are, however, important facts about the diagnosis and treatment of SCC in the breast that must be appreciated by surgeons. On a personal level I felt a sense of humiliation that this notification came from outside our profession as it suggested that we cannot monitor our own complications. Indeed we can do better, and we should use the announcement about SCC as an impetus to put systems in place to identify other plastic surgery complications in the future. This will be no easy task as there are many obstacles senselessly blocking our path, and we must endeavor to act in concert to dismantle these barriers.

The key messages from the notification were that fewer than 20 cases were documented—19 to be exact—a number so low that it did not rise to a level that required require a regulatory change. On March 8, 2023, the FDA sent out an updated announcement that did not document new cases but offered case details, as well as instructions for reporting cases.^2^ The first report of a breast implant–associated SCC was in 1992,^3^ and as of February 2023 an additional 10 to 20 cases worldwide had been identified (the number is inexact because the data are incomplete and there may be duplicate reports).^4-12^ Because SCC around breast implants was virtually unheard of by plastic surgeons and is a very serious disease, it was appropriate for the FDA to make that announcement. The disease is not unique to breast implants, occurring with materially different devices implanted in various anatomic regions of the body, including dental implants, a retained bullet fragment, and orthopedic joints.^13^ It has even been reported as a primary disease of the breast with no previous surgery.^14^

Unlike promptly diagnosed and treated cases of breast implant–associated anaplastic large cell lymphoma (BIA-ALCL), SCC of the breast implant capsule is highly aggressive and despite early intervention has nearly a 50% 6-month mortality rate.^1,15-17^ It is critical that future cases of SCC promptly be reported to the implant manufacturer and the FDA at www.accessdata.fda.gov.

Although not forewarned of either announcement, The Aesthetic Society promptly informed its members how breast implant–associated SCC should be diagnosed and treated, as well as how it could be discussed with patients.^17^ Presentation most often is sternal/chest pain, but also mass, seroma, swelling, erythema, and capsular contracture. Work-up involves an ultrasound to evaluate for periprosthetic fluid; aspiration of any such fluid, and in addition to tests to rule out BIA-ALCL, analysis of fluid for CK 5/6, p63, and flow cytometry for squamous cells and keratin. If positive, an MRI can be done both with and without contrast to evaluate for a mass, and a positron emission tomography/computed tomography scan can be ordered to ascertain the extent of the disease. Treatment consists of en bloc capsulectomy and complete resection of the tumor, which often extends beyond the capsule at the time of diagnosis. (Although there appears to be no role for an en bloc capsulectomy in the treatment of “breast implant illness,” it is an important part of an operation for a malignancy such as SCC.^18^) Chemotherapy and radiation seem ineffective. These can be very problematic cases and probably should be treated at major institutions prepared for what can be major resections of the chest wall and aggressive disease progression.

What should prospective and existing implant patients be told? It is simple enough to tell prospective patients there have been a couple of dozen cases worldwide out of the millions of women who have received breast implants. But if you are making a statement that simultaneously dismisses the risk as irrelevantly small, why bother to state it? My usual habit has not been to specifically discuss SCC, feeling the information presented to patients during a consultation needs to be triaged to the important complications that occur more frequently and guide their decision about whether to receive implants. Were physicians to discuss every potential complication of a drug or device that occurred with the frequency of SCC, they would only be able to see a patient or two in a day.

This does not change what existing implant patients should already know about cancers in the breast, namely that they should routinely perform self-exams, have regular mammograms, and immediately report any changes in their breasts to their plastic surgeons. The small additional risk of SCC to the other risks of breast implants would not in itself logically justify removal of an implant from most breast implant patients otherwise wanting to keep them. But for patients already considering removal of their implant, this may create enough additional concern that it tips the balance in favor of removal.

If one were thoroughly discussing the risk of breast cancer and breast implants, it would be necessary to mention that the preponderance of evidence indicates implants reduce the risk of invasive breast cancer, and that a miniscule risk reduction of this common cancer would far exceed the additive risk of SCC.^19-22^ Surgeons are loathe to discuss that implants might reduce the risk of breast cancer, perhaps because they do not know about the data, do not feel it is yet conclusive (it isn’t), or that it is distasteful to sound as if they are encouraging patients to get implants. But if the discussion topic is how breast implants are likely to affect the chance of developing breast cancer, then it would be logical to make this part of the discussion.

The mean time for presentation of BIA-ALCL is approximately 10 years after implantation, but is nearly 23 years for SCC. Whereas all BIA-ALCL cases in which the implant type was known had a textured surface, SCC has been observed regardless of material, fill, surface, or manufacturer. There is 1 reported case of SCC following injection of free silicone into the breast.^23^ Many of the cases are with manufacturers no longer in business or with implants never available in the United States. There are not enough cases to ascertain whether there are confounding variables such as incision, irrigation, or pocket location—or if changes in current techniques and the newest generation of implants matter. But given that SCC occurs with such a variety of implanted devices throughout the body I suspect that these issues do not substantially affect the risk.

In the hopes of exploring these potentially important confounding issues I contacted senior management at Mentor (Santa Barbara, CA), Allergan (Irvine, CA), and Sientra (Irvine, CA), telling them I was writing this paper and asking for their detailed SCC data. But I was unsuccessful, because none provided all the data I requested.

Of the 3 manufacturers, 1 promptly responded to say it had no cases in the United States but declined to offer information about its cases outside the United States. Another gave a spreadsheet listing partial data on its 4 cases—although I am aware of another case from several years ago in my community with their implant, so its dataset was both incomplete and did not contain the granular information I requested. This manufacturer should have accumulated all that data and they should have made it available. A third dillydallied and delayed for many weeks, with various factions in the company arguing for, and others against releasing the data, finally making the excuse that “releasing this data would set a bad precedent.” Yes, you read that correctly: the company did not want to put itself in a situation in which it would be expected to give cancer-related data on its products in the future. After a lot of pressure from me and upon hearing the other manufacturers had released their data, the manufacturer finally told me it had 1 case, with another under investigation, and offered no specific data on those cases. If we cannot get all of these data, then our ability to understand this disease is severely thwarted.

It is worth noting that even if each manufacturer had complied with my request, each had too few cases from which to make a valid comparison of risk between them. They also have never been willing to make sales figures available, so even if they told me the details of the cases, no rate would be calculable.

This problem is not new, and we should no longer accept it. Manufacturers have long obfuscated legitimate efforts by surgeons to honorably examine safety data, even though these data are the product of procedures performed by surgeons, reported by surgeons, and necessary to the decision-making of patients and doctors. Contracts between investigators and manufacturers for premarket approval trials forbid participating surgeons from independently publishing any data from the studies. Our societies must collectively insist that all such data must be made transparent and immediately available to all who are interested. Perhaps this effort can be made in concert with the FDA. If this is not successful, we should pursue legislation to require making these data available. The late John Tebbetts, MD, commented to me in an email, “Surgeons who contribute to premarket approval studies deserve access to their own personal outcome data as well as that of the entire study. So too do surgeons who use the devices and patients who receive them” (personal communication, August 2012).

I communicated directly with the FDA and requested its files on SCC, and was given a link to search for complications on its website (MAUDE, Manufacturer and User Facility Device Experience (https://www.accessdata.fda.gov/scripts/cdrh/cfdocs/cfmaude/search.cfm)). If the FDA had already evaluated the data, why should I have to go through that link rather than just reading its summary of the reported cases? You would think that they would be glad to have the expertise of a plastic surgeon looking into these cases and that they would eagerly send the files and ask for whatever analysis i could offer. I tried searching the database, but even knowing what to look for I could not identify the 19 cases of SCC, and so that renders looking for something otherwise unknown obviously impossible. Furthermore, the FDA's notification was distributed without previous consultation with the plastic surgery societies. In the future, the FDA should know that The Aesthetic Society and the Aesthetic Surgery Education and Research Foundation (ASERF) would be eager to share information, analyze data, and draft public notices together. We seem too often to be treated as not even a mere “stakeholder,” but one with such biases that we cannot be relied upon to be helpful. That is obviously false, and that insinuation deprives the public of the benefit of input from the individuals with the most expertise about breast implants and most vested in the welfare of breast implant patients. To wit, at a recent FDA hearing multiple anti-implant “activists” were given longer time for their presentations than any plastic surgeon expert. At an earlier hearing, manufacturers and the FDA ludicrously discussed the risk of scalpel injuries to breast implants while surgeons—not given a place at the podium—squirmed in their seats because they all know that a scalpel is only used in a breast augmentation to make the initial incision.

I have spoken with 2 surgeons trying to put together a comprehensive list of cases and analyze all the SCC data. They have different case totals, and the FDA has a third. Their efforts have been thwarted by the lawyers at academic institutions who frequently cite the Health Insurance Portability and Accountability Act as preventing them from offering deidentified patient information. Even when shown why this was incorrect, many hospital and university attorneys obfuscated their efforts with other capricious excuses. Are these lawyers merely incompetent? Or are they protecting the data so that their own institution can have lead authorship on a publication? Are the surgeons collaborating with full cooperation or are they holding back to maintain authorship or to nurture a special personal relationship with the FDA? Academic surgeons—of all people—should be the first to understand that data collected at their institution contribute to patient safety and should demand of themselves and their attorneys to find a way to collaborate for the sake of patient safety.

It is impossible for surgeons to routinely peruse the FDA database for problems we do not know exist. It is also impossible for any surgeon to read journals as remote from our specialty as the *International Journal of Surgical Pathology*, *Cancer*, or a non-peer-reviewed article in Research Square (Durham, NC) to even realize that there could be a problem. Instead of launching ChatGPT (OpenAI, San Francisco, CA) on a database that best serves the interest of plagiarizing college students, it would have been wiser were it to have been launched to examine the data of the world's medical journals. That or a similar technology should be given access to the FDA's complication reports and all the published articles hidden behind paywalls. This technology is available today and this relatively simple process should be encouraged by medical organizations and enforced by legislators if individuals or organizations stand in the way of this progress.

Meanwhile, editors should more often “dual publish” papers of interest to multiple specialties. For instance, the first publication of a US Brazilian butt lift death was in the *Journal of Forensic Sciences*.^24^ Publications about breast implants and autoimmune diseases have been published in a variety of rheumatology journals that no surgeon reads. Putting aside the prohibitive cost of buying such articles from unsubscribed journals to see if the article is even relevant, how would rheumatologists know about articles in plastic surgery journals and how would plastic surgeons know about articles in rheumatology journals? These papers—and those in the future—should have appeared in both plastic surgery journals and in journals of the specialty of their authors.

The major national societies have put great effort into creating registries and encouraging members to use them. But these are planting seeds that will not yield fruit for many years; for SCC that would be an average of 23 years, and who knows if the data collection beginning now will be functional at that time. Much easier to obtain—and much more valuable at identifying problems today—would be to focus on reporting problems as they are recognized. You’ve seen complications of devices, but how many times have you personally reported them to the manufacturer or the FDA? Do you even know how to do it? Professional medical societies should divert the substantial resources now being put toward creating prospective registries into creating a system for easy reporting and searching of complications as they arise. These societies should liaise with the FDA to compel industry to reveal the data on the devices we use. Registries are a noble concept, but our priority should be on identifying current problems. And there is little point in entering patients into a registry today if we do not reliably know that the systems are in place to report their complications tomorrow.

Although SCC of the implant capsule is very rare, it is “100% common” for the people who have it. The FDA should be commended for recognizing this life-threatening problem and bringing it to our attention. However, it is most disconcerting that our specialty was not the one to pick up on it. If we did not learn that lesson from our slow recognition of the Brazilian butt lift death risk, then we should learn now that we must put systems in place to recognize when a procedure is associated with serious complications. Hopefully some of the suggestions in this article can be implemented for the sake of our patients.
